# Postprandial effect of breakfast glycaemic index on vascular function, glycaemic control and cognitive performance (BGI study): study protocol for a randomised crossover trial

**DOI:** 10.1186/s13063-016-1649-x

**Published:** 2016-10-24

**Authors:** Natalia Sanchez-Aguadero, Luis Garcia-Ortiz, Maria C. Patino-Alonso, Sara Mora-Simon, Manuel A. Gomez-Marcos, Rosario Alonso-Dominguez, Benigna Sanchez-Salgado, Jose I. Recio-Rodriguez

**Affiliations:** 1 Primary Care Research Unit, The Alamedilla Health Centre, Castilla and León Health Service (SACYL), Institute of Biomedical Research of Salamanca (IBSAL), Salamanca, Spain; 2 Department of Biomedical and Diagnostic Sciences, University of Salamanca, Salamanca, Spain; 3 Department of Statistics, University of Salamanca, Salamanca, Spain; 4 Primary Care Research Unit, The Alamedilla Health Centre, Institute of Biomedical Research of Salamanca (IBSAL), Salamanca, Spain; 5 School of Labor Relations, University of Salamanca Affiliated Centre, Zamora, Spain; 6 Department of Medicine, University of Salamanca, Salamanca, Spain; 7 Department of Nursing and Physiotherapy, University of Salamanca, Salamanca, Spain; 8 Primary Care Research Unit, The Alamedilla Health Centre, Av. Comuneros N° 27, 37003 Salamanca, Spain

**Keywords:** Glycaemic index, Postprandial period, Vascular stiffness, Blood glucose, Cognition

## Abstract

**Background:**

Postprandial glycaemic response affects cognitive and vascular function. The acute effect of breakfast glycaemic index on vascular parameters is not sufficiently known. Also, the influence of breakfasts with different glycaemic index on cognitive performance has been mostly studied in children and adolescents with varying results. Therefore, the purpose of this study is to analyse the postprandial effect of high and low glycaemic index breakfasts on vascular function and cognitive performance and their relationship with postprandial glycaemic response in healthy young adults.

**Methods/design:**

This is a crossover clinical trial targeting adults (aged 20–40 years, free from cardiovascular disease) selected by consecutive sampling at urban primary care health clinics in Salamanca (Spain). Each subject will complete three interventions with a washout period of one week: a control condition (consisting of water); a low glycaemic index breakfast (consisting of dark chocolate, walnuts, yogurt and an apple, with an overall glycaemic index of 29.4 and an energy contribution of 1489 kJ); and a high glycaemic index breakfast (consisting of bread, grape juice and strawberry jam, with an overall glycaemic index of 64.0 and an energy contribution of 1318 kJ). The postprandial effect will be assessed at 60 and 120 minutes from each breakfast including blood sampling and cognitive performance evaluations. Measurements of arterial stiffness and central haemodynamic parameters will be taken at –10, 0, 15, 30, 45, 60, 75, 90, 105 and 120 minutes.

**Discussion:**

The differences in postprandial glycaemic response due to breakfast glycaemic index could affect vascular parameters and cognitive performance with important applications and implications for the general population. This could provide necessary information for the establishment of new strategies in terms of nutritional education and work performance improvement.

**Trial registration:**

ClinicalTrials.gov: ﻿NCT02616276﻿. Registered on 19 November 2015.

**Electronic supplementary material:**

The online version of this article (doi:10.1186/s13063-016-1649-x) contains supplementary material, which is available to authorized users.

## Background

The glycaemic index (GI) is a measure of the speed with which a carbohydrate is absorbed compared to a reference product (pure glucose) [1, 2]. Diets with a high GI increase the risk of diseases related to lifestyles such as type 2 diabetes mellitus [3, 4]. A recent meta-analysis of 14 prospective studies found that high GI diets are associated with an increased risk of cardiovascular disease (CVD) [5], while a reduction in dietary GI can favourably affect the incidence of coronary disease in women [6]. Low GI diets might reduce the risk of CVD because they decrease postprandial glycaemia with different metabolic effects including differences in insulin sensitivity, circulating lipid concentrations and vascular function [3].

Regarding this latter aspect, the currently accepted gold standard to assess arterial stiffness is the carotid-femoral pulse wave velocity (PWV) [7], which has been related to increased morbidity and mortality in both patients with CVD and healthy individuals [8, 9]. Likewise, the augmentation index (AIx) is a measure of wave reflection and arterial stiffness that has been shown to be a predictor of both future cardiovascular events and all-cause mortality [10]. In this way, the Lifestyles and Vascular Aging (EVIDENT) study [11] analysed the relationship between lifestyle and arterial aging in a sample of 1553 subjects who were free from CVD. We concluded that low GI diets were associated with lower AIx values. In this regard, a reduction in central haemodynamic parameters, AIx and PWV, at 60 minutes from food intake has been reported in healthy adults, perhaps because of an increase in insulin and/or visceral vasodilatation [12]. Another possible explanation for these findings might be the postprandial hypotension that occurs after a meal due to decreased cortisol secretion and activation of the parasympathetic system [13]. For these reasons, although the effects of various macronutrients on vascular function have been explored in a number of studies [14–17], Taylor et al. [12] underlined the importance of analysing the impact of different types of meals on parasympathetic activity, central blood pressure (CBP) and vascular function parameters.

Of particular interest is the carbohydrate (CHO) content of a meal, which changes postprandial glucose and insulin levels and results in varying AIx reductions in postmenopausal women [18]. Thus, breakfast would play a fundamental role, because traditional breakfast foods tend to be high in CHOs, and there can be variability in the GI of these CHOs. It appears that consuming food early in the day can have a beneficial metabolic impact regardless of GI and that low GI meals can be of more value for glycaemic control in the morning than the evening [19]. However, despite the fact that breakfast patterns are associated with metabolic profiles [20], few authors have studied their effect on cardiovascular responses. Ahuja et al. [21] found that a light breakfast (1301 kJ energy) reduced AIx, CBP and blood pressure (BP), and increased heart rate (HR) in adults versus fasting (water). In contrast, a trial aimed to compare the dietary effects of a high GI with a low GI breakfast replacement in obese and overweight individuals reported no differences in BP or insulin concentration between breakfasts together with beneficial changes in fasting glucose after the consumption of a low GI breakfast [22].

On the other hand, increasing evidence has shown that the postprandial glycaemic response also has a potential impact on cognitive function [23]. Due to the importance that cognitive processes have on professional development, there is interest in examining the influence of breakfasts with different GI values on cognitive outcomes in healthy young adults.

Cognitive performance may be influenced by many factors including individual and socioeconomic differences or nutritional and health status [24]. The effect that breakfasts with different GIs may have on cognitive performance has been studied in people with type 2 diabetes mellitus and obesity, but it is not clear that a specific GI breakfast could benefit cognitive processes in these participants [23, 25]. However, various studies conducted on children have explored the relationship between breakfasts consisting of different GI foods and cognitive functions with contrasting results [24, 26–29]. It appears that a low GI breakfast can benefit the immediate [28–30] and delayed [31] verbal memory as well as sustained attention [30] and verbal fluency [32]. A high GI breakfast may confer benefits for selective attention, processing speed and working memory [32]. In addition, consuming different GI carbohydrates at breakfast could modulate cognitive performance, but this effect requires more study [33].

### Objectives

The present study aims to evaluate, in a sample of healthy young adults, the postprandial effect of low and high glycaemic index (GI) breakfasts on vascular function, as measured by central blood pressure, augmentation index and pulse wave velocity and also cognitive performance. The secondary goal is to analyse the association of postprandial glycaemic response with vascular function and cognitive performance for high versus low GI breakfasts.

## Methods/design

### Design and setting

**Fig. 1:**
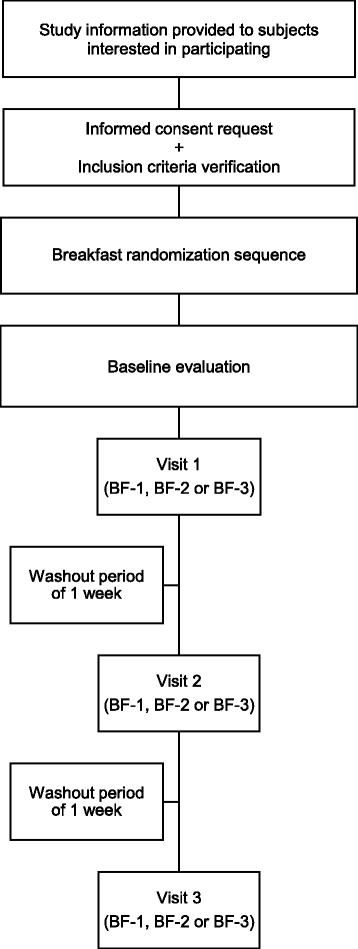
Flowchart of breakfast glycaemic index study. *BF* breakfast

We designed a controlled crossover clinical trial where each subject will complete three interventions (control condition, high GI breakfast and low GI breakfast) with a washout period of one week between each trial. The order will be determined by a randomisation sequence generated using randomization.com software (http://www.randomization.com) (Fig. 1). The SPIRIT checklist is provided as Additional file 1.

#### Study setting

The study will be conducted in the primary care health area of Salamanca in “La Alamedilla” Research Unit belonging to the Spanish Network for Preventive Activities and Health Promotion (redIAPP) and the Institute of Biomedical Research of Salamanca (IBSAL).

### Study population

The subjects will be selected by consecutive sampling in the primary care clinics of urban health centres from Salamanca (Spain) between 2015 and 2016.

#### Inclusion criteria

The study targets young adults aged 20–40 years of both sexes who agree to sign the informed consent document.

#### Exclusion criteria

Subjects will be excluded who have a history of cardiovascular events (acute myocardial infarction, stroke, etc.), hypertension, diabetes mellitus, dyslipidaemia, pharmacological treatment for any of these conditions, neurological and/or neuropsychological disease or the consumption of toxic substances. We will also exclude patients with celiac disease and/or those intolerant to lactose, subjects on a low-calorie and/or low-sodium diet, pregnant women or those with any other circumstance that the investigators suggest will interfere with the study procedures.

### Sample size

The primary outcome variable is change in central augmentation index (AIx). The Conduit Artery Function Evaluation (CAFE) study [8] found a reduction in cardiovascular events associated with a decline of 6.5 (5.8, 7.3) points in the AIx. This is the basis of our calculation. The power calculation was a repeated measures design and compared both intervention breakfasts with a control condition with an alpha risk of 0.05 and a beta risk of 0.2. The standard deviation (SD) was 10 with a correlation coefficient between the initial and final measurement of 0.7. Thus, 40 subjects are required to detect a minimum difference of 5 points in the AIx between two intervention breakfasts. A loss to follow-up of 5 % was estimated.

### Study procedures

**Fig. 2:**
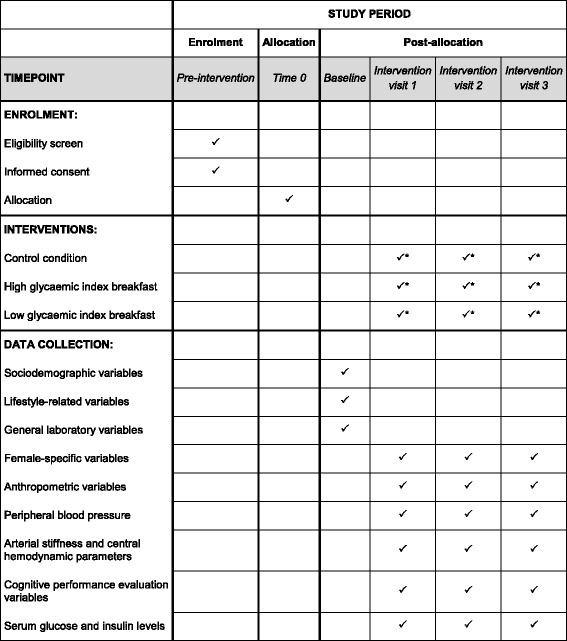
SPIRIT diagram. * Randomly assigned breakfast

On arrival at the research unit, subjects will be weighed, and their height, waist circumference and hip circumference will be measured. Participants will be seated and remain in this position throughout the visit. After 5 minutes of rest, a peripheral blood pressure measurement will be performed, and immediately the central blood pressure and haemodynamic parameters will be obtained. Next, cognitive performance will be assessed, fasting blood samples will be collected and central blood pressure and haemodynamic parameters will be determined again. Subjects will be provided with a randomly assigned breakfast to be consumed within 10 minutes. At the first bite, a timer will be started and additional measurements of central blood pressure and haemodynamic parameters will be taken every 15 minutes. Furthermore, another two cognitive performance evaluations and postprandial blood sampling will be completed at 60 and 120 minutes. Figure 2 shows the Standard Protocol Items: Recommendations for Interventional Trials (SPIRIT) diagram for the trial procedure.

### Intervention

Each of the three scheduled visits will last 2 hours, 40 minutes; this will occur between 8:15 am and 10:55 am. Participants will be asked to fast for 12 hours overnight prior and to limit their physical activity, alcohol consumption and smoking during the previous 24–48 hours.

#### Nutritional composition of each intervention arm

The nutritional composition of each intervention arm is described as follows:

1. Control condition (BF-1):
  This will consist of 350 mL of water served at room temperature.
2. High glycaemic index breakfast (BF-2):
  This will consist of 350 mL of water served at room temperature, 200 mL of grape juice (with 569 kJ/136 kcal), 40 g of white bread (2 slices of 218 kJ/52 kcal each) and 29 g of strawberry jam (with 313 kJ/75 kcal), providing a total of 1318 kJ/315 kcal. The nutrient composition of this meal is 72.0 g (91.4 %) carbohydrate, 0.9 g (2.6 %) fat, 3.9 g (5 %) protein and 1.6 g (1 %) fibre, with an overall GI of 64.0.
3. Low glycaemic index breakfast (BF-3):
  This will consist of 350 mL of water served at room temperature, a 150-g apple (with 339 kJ/81 kcal), a 125-g low-fat natural yogurt (with 234 kJ/56 kcal), 3 shelled walnuts (with 163 kJ/39 kcal per unit) and 17.5 g of 72 % dark chocolate (with 427 kJ/102 kcal), supplying a total of 1489 kJ/356 kcal. The nutrient composition of this meal is 31.5 g (35.4 %) carbohydrate, 19.9 g (50.3 %) fat, 9.7 g (10.9 %) protein and 6.0 g (3.4 %) fibre, with an overall GI of 29.4.

### Blinding strategy

Because of the nature of the interventions, participants and research staff cannot be blinded. However, those responsible for statistical analysis will be blinded to the interventions.

### Variables and measurement instruments

#### Sociodemographic variables

At the time of study entry and prior to the first intervention visit, data on age, gender, marital status, educational level and occupation will be collected.

#### Lifestyle-related variables

The following lifestyle-related variables will be collected at the time of study entry and prior to the first intervention visit:

1. Smoking:
  This will be measured using a questionnaire on smoking history and tobacco consumption pattern.
2. Alcohol consumption:
  This will be measured using a questionnaire on alcohol consumption in the past 7 days specifying drinks and their volumes.
3. Regular diet:
  This will be assessed with the Diet Quality Index (DQI) [34], which is a validated questionnaire that records the frequency of food intake and assigns a score ranging from 18 to 54 points.
4. Regular physical activity:
  This will be evaluated with the short version of the International Physical Activity Questionnaire (IPAQ) [35], validated for a Spanish population. It assesses activity in the last 7 days differentiating between three types (walking, moderate-intensity and vigorous-intensity activity), according to the energy expenditure estimated for each of them (3.3, 4 and 8 metabolic equivalents of task [MET], respectively). It allows the MET-minutes/week to be calculated and subjects to be classified according to three activity levels (low, intermediate and high).

#### Female-specific variables

The date of the last menstruation will be recorded, because it may impact the study variables.

#### Anthropometric variables

The height will be measured, with the subject standing barefoot, to record the average of two readings rounded to the nearest centimetre using a portable system (Seca 222; Medical scale and measurement system, Birmingham, UK).

The body weight will be measured, with the subject barefoot and wearing light clothing, to record the average of two readings rounded to 100 g using a standard electronic balance (Seca 770; Medical scale and measurement system, Birmingham, UK) that is properly calibrated (precision ± 0.1 kg).

Following the recommendations of the Spanish Society for the Study of Obesity (SEEDO) [36], the waist circumference will be measured in duplicate at the level of the midpoint between the last rib and the iliac crest (with the subject standing without clothing) using a flexible tape parallel to the floor after inspiration. Hip circumference will be similarly measured at the level of the trochanters.

#### Peripheral blood pressure

Three measurements of systolic (SBP) and diastolic (DBP) blood pressure will be performed using the average of the last two with a validated Omron M10-IT model sphygmomanometer (Omron Healthcare, Kyoto, Japan). The measurements will be made on the participant’s dominant arm in a seated position after at least 5 minutes of rest with a cuff of appropriate size as determined by measurement of the upper arm circumference and following the recommendations of the European Society of Hypertension [37].

#### Arterial stiffness and central haemodynamic parameters

The Mobil-O-Graph® device [38] will be used to estimate cardiac output and total peripheral vascular resistance and to measure central blood pressure (CBP), pulse wave velocity (PWV), reflection coefficient and augmentation index (AIx). This is affected by heart rate (HR), so its values will be corrected to an HR of 75 bpm. This device will be scheduled to perform continuous measurements at –10, 0, 15, 30, 45, 60, 75, 90, 105 and 120 minutes with the subject sitting and resting his arm on a rigid surface.

#### Laboratory variables

At the time of study entry and prior to the first intervention visit, fasting plasma creatinine, serum total cholesterol, high-density lipoprotein (HDL) cholesterol, low-density lipoprotein (LDL) cholesterol and triglyceride values will be determined using standard enzymatic automated methods.

During each study visit, three cannula blood samples will be collected at –10, 60 and 120 minutes to measure serum glucose and insulin levels by ultraviolet-visible spectrophotometry and chemiluminescence, respectively. Serum will be isolated by centrifugation and stored in a –20 °C freezer within 48–72 hours until analysis. Samples will be treated and centrifuged by a single researcher under standardised conditions. The analysis will be performed in a laboratory in external quality assurance programs of the Spanish Society of Clinical Chemistry and Molecular Pathology.

#### Cognitive performance evaluation variables

For each of the three visits and for each evaluation within the same visit, a list of 15 different words from the Rey Auditory Verbal Learning Test and its alternative versions [39–41] will be used to evaluate the immediate verbal memory via immediate recall over three attempts. Delayed verbal memory will be assessed by free recall of the words learnt in the first part of the evaluation after a period of 10 minutes. Phonological fluency will be explored by enumerating for one minute as many words as possible starting with different letters [42]. The Trail Making Test A will be used to assess attention and processing speed, while executive functions will be explored using the Trail Making Test B [43]. Working memory will be traced with the WAIS Digit Span Backward test [44]. Finally, sustained and selective attention, executive functions and processing speed will be explored with the Stroop test [45].

### Statistical analysis

The normal distribution of variables will be verified using a Kolmogorov-Smirnov test. Quantitative variables will be displayed as the mean ± standard deviation if normally distributed or as the median (interquartile range) if asymmetrically distributed. The data will be quantitated using the repeated measures for analysis of variance (ANOVA) or the Friedman test if the data are non-normally distributed. To compare the differences among the three types of breakfast, the ANOVA test will be used, or the Wilcoxon test if the data are non-normally distributed. We will use the least significant difference (LSD) test as post hoc analysis. The relationship of quantitative variables to each other will be tested using Pearson’s or Spearman’s correlation as appropriate. The effect of the interventions can be modified by conditions such as the last menstruation date. To control the effect of confounders on the study results and to adequately evaluate the effect of the interventions, a multivariate analysis will be performed using the general linear model (GLM) in basic or extended models. The contrasting hypothesis will establish an alpha risk of 0.05 as the limit of statistical significance. The data will be analysed using IBM’s SPSS Statistics for Windows version 23.0 (IBM Corporation, Armonk, NY, USA).

### Methodological limitations

This study follows all the Consolidated Standards of Reporting Trials (CONSORT) recommendations, but participants cannot be blinded due to the intervention characteristics. However, the investigator who analyses the data will be blinded.

The acute effect of the high or low glycaemic index breakfast on the study variables may be influenced by conditions that cause endothelial dysfunction such as hypertension, diabetes or dyslipidaemia. Therefore, subjects with CVD, hypertension, diabetes or dyslipidaemia will be excluded.

Attempts to standardise food intake prior to experimental trials will not be made. However, participants will be asked to maintain stable dietary habits.

## Discussion

There is accumulating evidence that postprandial glycaemic response is associated with cognitive and vascular function [18, 23]. To our knowledge, the postprandial effects of high and low glycaemic index (GI) breakfasts on vascular parameters and cognitive performance have not been previously concurrently investigated.

Contrasting results have been reported about the effect of breakfast on vascular function and haemodynamic parameters [21, 22]. In this project, we expect to demonstrate that the lower glycaemic response to a low GI breakfast will have a greater lowering effect on measures of vascular function relative to a high GI breakfast.

Secondly, based on the findings of previous studies conducted on children and adolescents [26–30, 32], we hypothesise that we will see similar effects in healthy young adults. Thus, we expect that the lower glycaemic response to a low GI breakfast will have a positive impact on immediate and delayed verbal memory and verbal fluency, while the corresponding higher glycaemic response to a high GI breakfast will positively affect attention, processing speed and working memory.

Therefore, according to our hypothesis, the results of the current study may explain the influence of GI on cognitive and vascular function. Moreover, given the high worldwide prevalence of cardiovascular disease and its close relationship with cognitive decline, it would be interesting to know how the vascular parameters and cognitive processes are affected by the type of breakfast consumed. This could be employed for the design of novel lifestyle and dietary interventions.

Finally, our results may provide tools for adapting breakfast composition to the type of task to be performed and consequently for improving work performance.

## Trial status

This trial is currently recruiting participants.

## Additional file

Additional file 1: Populated SPIRIT checklist. (PDF 55 kb)

## Abbreviations

AIx: Augmentation index
BF: Breakfast
BGI: Breakfast glycaemic index
BP: Blood pressure
CAFE: Conduit Artery Function Evaluation
CBP: Central blood pressure
CHO: Carbohydrate
CREC: Clinical research ethics committee
CVD: Cardiovascular disease
DBP: Diastolic blood pressure
DQI: Diet Quality Index
EVIDENT: Lifestyles and Vascular Aging (study)
GI: Glycaemic index
HR: Heart rate
IBSAL: Institute of Biomedical Research of Salamanca
IPAQ: International Physical Activity Questionnaire
LOPD: Legislation on personal data protection
MET: Metabolic equivalent of task
PWV: Pulse wave velocity
REDIAPP: Network for preventive activities and health promotion
SBP: Systolic blood pressure
SEEDO: Spanish Society for the Study of Obesity
WAIS: Wechsler Adult Intelligence Scale

## References

[1] Jenkins DJ, Wolever TM, Taylor RH, Barker H, Fielden H, Baldwin JM, Bowling AC, Newman HC, Jenkins AL, Goff DV (1981). Glycemic index of foods: a physiological basis for carbohydrate exchange. Am J Clin Nutr.

[2] Foster-Powell K, Holt SH, Brand-Miller JC (2002). International table of glycemic index and glycemic load values: 2002. Am J Clin Nutr.

[3] Barclay AW, Petocz P, McMillan-Price J, Flood VM, Prvan T, Mitchell P, Brand-Miller JC (2008). Glycemic index, glycemic load, and chronic disease risk—a meta-analysis of observational studies. Am J Clin Nutr.

[4] Greenwood DC, Threapleton DE, Evans CE, Cleghorn CL, Nykjaer C, Woodhead C, Burley VJ (2013). Glycemic index, glycemic load, carbohydrates, and type 2 diabetes: systematic review and dose-response meta-analysis of prospective studies. Diabetes Care.

[5] Ma XY, Liu JP, Song ZY (2012). Glycemic load, glycemic index and risk of cardiovascular diseases: meta-analyses of prospective studies. Atherosclerosis.

[6] Mirrahimi A, de Souza RJ, Chiavaroli L, Sievenpiper JL, Beyene J, Hanley AJ, Augustin LS, Kendall CW, Jenkins DJ (2012). Associations of glycemic index and load with coronary heart disease events: a systematic review and meta-analysis of prospective cohorts. J Am Heart Assoc.

[7] Laurent S, Cockcroft J, Van Bortel L, Boutouyrie P, Giannattasio C, Hayoz D, Pannier B, Vlachopoulos C, Wilkinson I, Struijker-Boudier H (2006). Expert consensus document on arterial stiffness: methodological issues and clinical applications. Eur Heart J.

[8] Williams B, Lacy PS, Thom SM, Cruickshank K, Stanton A, Collier D, Hughes AD, Thurston H, O’Rourke M (2006). Differential impact of blood pressure-lowering drugs on central aortic pressure and clinical outcomes: principal results of the Conduit Artery Function Evaluation (CAFE) study. Circulation.

[9] Mattace-Raso FU, van der Cammen TJ, Hofman A, van Popele NM, Bos ML, Schalekamp MA, Asmar R, Reneman RS, Hoeks AP, Breteler MM (2006). Arterial stiffness and risk of coronary heart disease and stroke: the Rotterdam Study. Circulation.

[10] Vlachopoulos C, Aznaouridis K, O’Rourke MF, Safar ME, Baou K, Stefanadis C (2010). Prediction of cardiovascular events and all-cause mortality with central haemodynamics: a systematic review and meta-analysis. Eur Heart J.

[11] Recio-Rodriguez JI, Gomez-Marcos MA, Patino-Alonso MC, Rodrigo-De Pablo E, Cabrejas-Sanchez A, Arietaleanizbeaskoa MS, Repiso-Gento I, Gonzalez-Viejo N, Maderuelo-Fernandez JA, Agudo-Conde C (2015). Glycemic index, glycemic load, and pulse wave reflection in adults. Nutr Metab Cardiovasc Dis.

[12] Taylor JL, Curry TB, Matzek LJ, Joyner MJ, Casey DP (2014). Acute effects of a mixed meal on arterial stiffness and central hemodynamics in healthy adults. Am J Hypertens.

[13] Zanasi A, Tincani E, Evandri V, Giovanardi P, Bertolotti M, Rioli G (2012). Meal-induced blood pressure variation and cardiovascular mortality in ambulatory hypertensive elderly patients: preliminary results. J Hypertens.

[14] Lithander FE, Herlihy LK, Walsh DM, Burke E, Crowley V, Mahmud A (2013). Postprandial effect of dietary fat quantity and quality on arterial stiffness and wave reflection: a randomised controlled trial. Nutr J.

[15] Hall WL, Sanders KA, Sanders TA, Chowienczyk PJ (2008). A high-fat meal enriched with eicosapentaenoic acid reduces postprandial arterial stiffness measured by digital volume pulse analysis in healthy men. J Nutr.

[16] Giannattasio C, Zoppo A, Gentile G, Failla M, Capra A, Maggi FM, Catapano A, Mancia G (2005). Acute effect of high-fat meal on endothelial function in moderately dyslipidemic subjects. Arterioscler Thromb Vasc Biol.

[17] Papamichael CM, Karatzi KN, Papaioannou TG, Karatzis EN, Katsichti P, Sideris V, Zakopoulos N, Zampelas A, Lekakis JP (2008). Acute combined effects of olive oil and wine on pressure wave reflections: another beneficial influence of the Mediterranean diet antioxidants?. J Hypertens.

[18] Greenfield JR, Samaras K, Chisholm DJ, Campbell LV (2007). Effect of postprandial insulinemia and insulin resistance on measurement of arterial stiffness (augmentation index). Int J Cardiol.

[19] Gibbs M, Harrington D, Starkey S, Williams P, Hampton S (2014). Diurnal postprandial responses to low and high glycaemic index mixed meals. Clin Nutr (Edinburgh, Scotland).

[20] di Giuseppe R, Di Castelnuovo A, Melegari C, De Lucia F, Santimone I, Sciarretta A, Barisciano P, Persichillo M, De Curtis A, Zito F (2012). Typical breakfast food consumption and risk factors for cardiovascular disease in a large sample of Italian adults. Nutr Metab Cardiovasc Dis.

[21] Ahuja KD, Robertson IK, Ball MJ (2009). Acute effects of food on postprandial blood pressure and measures of arterial stiffness in healthy humans. Am J Clin Nutr.

[22] Pal S, Lim S, Egger G (2008). The effect of a low glycaemic index breakfast on blood glucose, insulin, lipid profiles, blood pressure, body weight, body composition and satiety in obese and overweight individuals: a pilot study. J Am Coll Nutr.

[23] Lamport DJ, Chadwick HK, Dye L, Mansfield MW, Lawton CL (2014). A low glycaemic load breakfast can attenuate cognitive impairments observed in middle aged obese females with impaired glucose tolerance. Nutr Metab Cardiovasc Dis.

[24] Edefonti V, Rosato V, Parpinel M, Nebbia G, Fiorica L, Fossali E, Ferraroni M, Decarli A, Agostoni C (2014). The effect of breakfast composition and energy contribution on cognitive and academic performance: a systematic review. Am J Clin Nutr.

[25] Lamport DJ, Dye L, Mansfield MW, Lawton CL (2013). Acute glycaemic load breakfast manipulations do not attenuate cognitive impairments in adults with type 2 diabetes. Clin Nutr (Edinburgh, Scotland).

[26] Micha R, Rogers PJ, Nelson M (2010). The glycaemic potency of breakfast and cognitive function in school children. Eur J Clin Nutr.

[27] Ingwersen J, Defeyter MA, Kennedy DO, Wesnes KA, Scholey AB (2007). A low glycaemic index breakfast cereal preferentially prevents children’s cognitive performance from declining throughout the morning. Appetite.

[28] Mahoney CR, Taylor HA, Kanarek RB, Samuel P (2005). Effect of breakfast composition on cognitive processes in elementary school children. Physiol Behav.

[29] Hoyland A, Dye L, Lawton CL (2009). A systematic review of the effect of breakfast on the cognitive performance of children and adolescents. Nutr Res Rev.

[30] Benton D, Maconie A, Williams C (2007). The influence of the glycaemic load of breakfast on the behaviour of children in school. Physiol Behav.

[31] Benton D, Ruffin MP, Lassel T, Nabb S, Messaoudi M, Vinoy S, Desor D, Lang V (2003). The delivery rate of dietary carbohydrates affects cognitive performance in both rats and humans. Psychopharmacology (Berl).

[32] Micha R, Rogers PJ, Nelson M (2011). Glycaemic index and glycaemic load of breakfast predict cognitive function and mood in school children: a randomised controlled trial. Br J Nutr.

[33] Papanikolaou Y, Palmer H, Binns MA, Jenkins DJ, Greenwood CE (2006). Better cognitive performance following a low-glycaemic-index compared with a high-glycaemic-index carbohydrate meal in adults with type 2 diabetes. Diabetologia.

[34] Schroder H, Benitez Arciniega A, Soler C, Covas MI, Baena-Diez JM, Marrugat J (2012). Validity of two short screeners for diet quality in time-limited settings. Public Health Nutr.

[35] Roman Vinas B, Ribas Barba L, Ngo J, Serra ML (2013). Validity of the international physical activity questionnaire in the Catalan population (Spain). Gac Sanit.

[36] Salas-Salvado J, Rubio MA, Barbany M, Moreno B (2007). SEEDO 2007 Consensus for the evaluation of overweight and obesity and the establishment of therapeutic intervention criteria. Med Clin.

[37] 2013 Practice guidelines for the management of arterial hypertension of the European Society of Hypertension (ESH) and the European Society of Cardiology (ESC): ESH/ESC Task Force for the Management of Arterial Hypertension. J Hypertens. 2013;31(10):1925–38. 10.1097/HJH.0b013e328364ca4c 24107724

[38] Weiss W, Gohlisch C, Harsch-Gladisch C, Tolle M, Zidek W, van der Giet M (2012). Oscillometric estimation of central blood pressure: validation of the Mobil-O-Graph in comparison with the SphygmoCor device. Blood Press Monit.

[39] Rey A (1964). L’Examen clinique en psychologie.

[40] Shapiro DM, Harrison DW (1990). Alternate forms of the AVLT: a procedure and test of form equivalency. Arch Clin Neuropsychol.

[41] Lezak MD (1983). Neuropsychological assessment.

[42] Valencia NJ, Laserna JA, Pérez-García M, Orozco C, Miñán M, Garrido C, Peralta I, Morente G (2000). Influencia de la escolaridad y el sexo sobre la ejecución en el FAS, nombrar animales y nombrar frutas. Psicol Conduct.

[43] Reitan RM (1992). Trail Making Test: manual for administration and scoring.

[44] Wechsler D (1987). Wechsler Memory Scale — Revised manual.

[45] Golden CJ (2005). Stroop: test de colores y palabras.

